# Promoted

**Published:** 1862-11

**Authors:** 


PROMOTED.—Dr. A. J. Steele, formerly resident physician and surgeon to the Buffalo General Hospital, and more recently, assistant surgeon to the 26th Regt., N. Y.V., has been appointed surgeon to the 38th Regt., N. Y,V.

Dr. Steele has been in active service since the commencement of the war, and will bring to the discharge of his duty a very ripe experience for a young surgeon. We most heartily congratulate the doctor upon his advancement and also the 38th regiment, in obtaining the services of one of the most energetic, intelligent and experienced surgeons.

